# Tinker, Tailor, Soldier, Spy: The Diverse Roles That Fluorine Can Play within Amino Acid Side Chains

**DOI:** 10.3390/molecules28176192

**Published:** 2023-08-22

**Authors:** Samantha A. Miles, Joshua Andrew Nillama, Luke Hunter

**Affiliations:** School of Chemistry, The University of New South Wales (UNSW), Sydney 2052, Australia

**Keywords:** amino acid side chain, fluorination, peptides, proteins

## Abstract

Side chain-fluorinated amino acids are useful tools in medicinal chemistry and protein science. In this review, we outline some general strategies for incorporating fluorine atom(s) into amino acid side chains and for elaborating such building blocks into more complex fluorinated peptides and proteins. We then describe the diverse benefits that fluorine can offer when located within amino acid side chains, including enabling ^19^F NMR and ^18^F PET imaging applications, enhancing pharmacokinetic properties, controlling molecular conformation, and optimizing target-binding.

## 1. Introduction

Fluorination has proven to be an exceptionally useful strategy in the development of small-molecule drugs and agrochemicals [1,2,3,4,5,6,7,8,9,10]. The presence of fluorine can confer a variety of advantages including enhanced resistance to metabolism, higher membrane permeability, more potent target-binding, and greater target selectivity. 

While the value of fluorine is now well-established in the context of small-molecule drugs, a major current trend in the pharmaceutical industry is towards “beyond rule of 5” compounds, i.e., bioactive compounds that lie outside of the physicochemical parameters that are commonly accepted to correlate with oral bioavailability [11,12,13,14,15,16,17,18]. Particularly notable amongst this new generation of pharmaceutical agents are peptides and proteins. 

Given the track record of fluorination in the context of small-molecule drugs, it seems likely that fluorination could offer significant benefits in the optimization of peptide- and protein-based drugs too.

The structure of a peptide offers several possible sites for fluorination, which can be broadly categorized as on the *backbone* or on the *side chain*. In terms of the peptide backbone, fluorine can be found within non-hydrolyzable amide isosteres (e.g., CF=CH; C(CF_3_)=CH; C(CF_3_)–NH), or partway along a backbone-extended amino acid (e.g., fluorostatines such as H_2_N–CH(^i^Bu)–CH(OH)–CF_2_–CO_2_H). We have recently reviewed some of these aspects of backbone fluorination [19].

**Figure 1:**
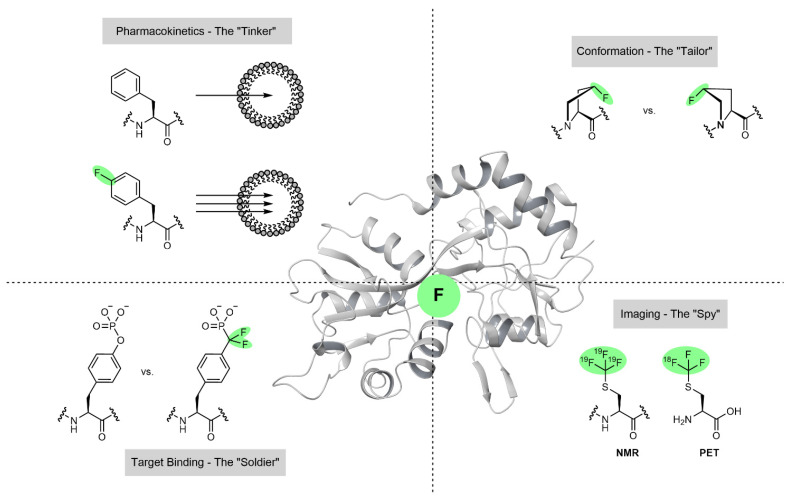
Fluorine can play diverse roles when located within amino acid side chains.

In the present review, we focus on *side chain* fluorination (Figure 1). We will briefly discuss the various strategies for synthesizing side chain-fluorinated amino acids (Section 2), and then we will delve into the varied roles that fluorine can play within amino acid side chains, including enabling NMR and PET imaging applications (Section 3); remediating problematic pharmacokinetic properties (Section 4); controlling conformation on scales ranging from individual amino acids all the way up to protein quaternary structure (Section 5); and, finally, enhancing target-binding interactions (Section 6). There have been several excellent reviews of some of these topics [20,21,22,23,24,25,26,27,28] but we feel that it is worthwhile to provide an updated and broad account of the field. 

## 2. Synthetic Aspects

Mirroring the structural diversity of side chain-fluorinated amino acids is a diversity of methods available for their chemical synthesis. Several reviews have already been published on this topic [29,30,31,32,33,34,35,36,37,38], so we will provide only a very brief overview here (Section 2.1). Additionally, we will provide a concise discussion of methods for elaborating side chain-fluorinated amino acids into peptides and proteins (Section 2.2).

### 2.1. Strategies for the Synthesis of Side Chain-Fluorinated Amino Acids 

**Scheme 1:**
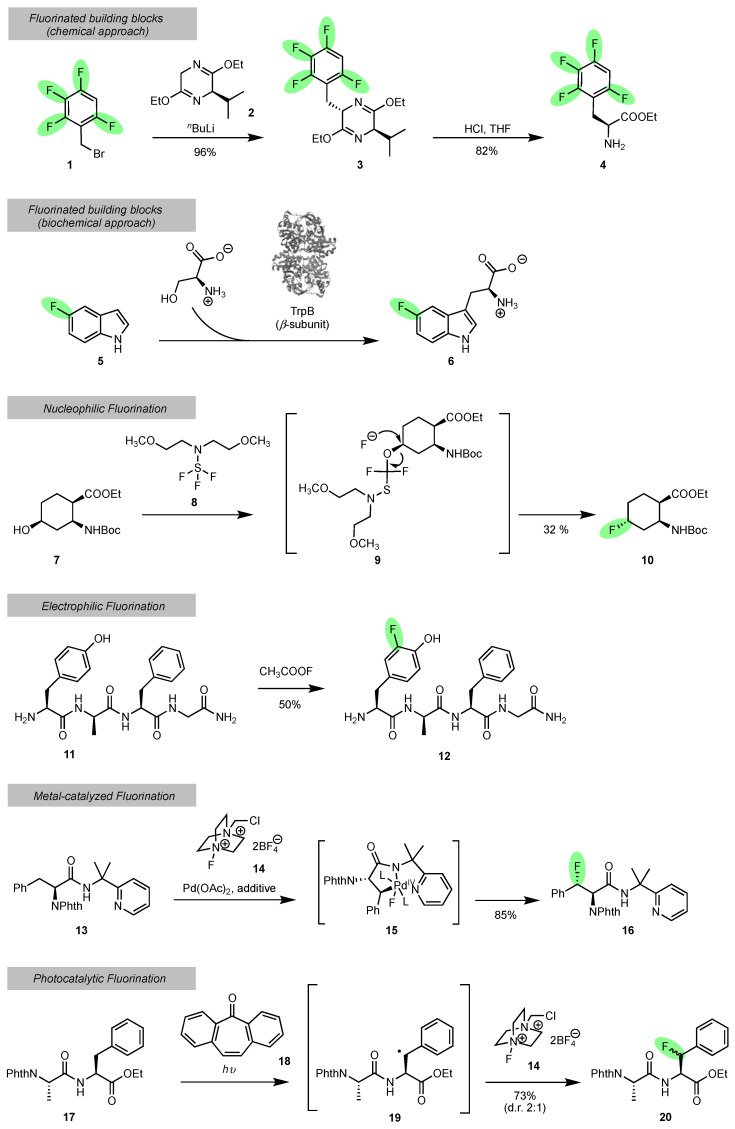
General strategies for synthesizing side chain-fluorinated amino acids.

One way to efficiently obtain a side chain-fluorinated amino acid is to commence with a simple commercially available *fluorinated building block* [39,40,41,42,43,44,45,46,47,48,49,50,51,52,53,54,55,56,57], and then append the required amino and carboxylate groups using one of several established methods. The Schöllkopf approach is a typical example (Scheme 1). By this means, the simple building block 2,3,4-6-tetrafluorobenzyl bromide (**1**) can be converted, stereoselectively, into 2,3,4,6-tetrafluorophenylalanine (**3**) [34,58,59].

*Biochemical methods* can also be utilized for transforming simple fluorinated building blocks into side chain-fluorinated amino acids [60,61,62]. For example, the directed evolution of tryptophan synthase β-subunit (TrpB) from *Pyrococcus furiosus* generated a mutant enzyme that could efficiently convert 5-fluoroindole (**5**) into the fluorinated tryptophan analog **6** [60].

A range of methods are available for forming the C–F bond at a later stage of the amino acid synthesis. Such fluorination methods can be broadly categorized according to the mechanism involved, one of which is *nucleophilic fluorination* [63,64,65,66,67,68,69,70,71,72]. A nucleophilic source of fluoride such as diethylaminosulfur trifluoride (DAST), morpholinosulfur trifluoride (morph-DAST), or silver fluoride (AgF) can be employed to displace a leaving group and deliver a side chain-fluorinated amino acid product. A typical example is shown in Scheme 1: treatment of the secondary alcohol **7** with Deoxo-Fluor (**8**) affected substitution with inversion to deliver the fluorinated β-amino ester **10** [67]. However, the modest yield of this particular transformation highlights a limitation of the nucleophilic fluorination approach more generally, which is that it can sometimes be outcompeted by side reactions such as elimination or rearrangement. 

Fluorine can alternatively be incorporated into amino acid side chains via *electrophilic fluorination*. Electrophilic sources of fluorine such as *N*-fluorobenzensulfonimide (NFSI) or Selectfluor (**14**) can be employed to react with electron-rich amino acid side chains and deliver fluorinated targets [73,74,75,76,77,78]. For example, the electron-rich tyrosine side chain (**11**) can undergo an S_E_Ar reaction with acetyl hypofluorite to deliver the fluorinated tyrosine derivative **12** in a reasonable yield (Scheme 1) [79]. This transformation is notable for its chemoselectivity: no reaction at the less electron-rich phenylalanine side chain of **11** is observed. 

Another method for the synthesis of side chain-fluorinated amino acids is *metal-catalyzed fluorination* [80,81]. For example, palladium catalysis has been applied for the site-selective fluorination of a non-functionalized sp^3^ carbon to produce the fluorinated α-amino acid derivative **16** (Scheme 1) [82]. This reaction was aided by the directing group PIP (2-(pyridin-2-yl)isopropyl amine) that facilitated metal coordination. 

Yet another general strategy for synthesizing side chain-fluorinated amino acids is *photocatalytic/radical fluorination* [83,84]. Using catalytic dibenzosuberenone (**18**) and Selectfluor (**14**) as the fluorine source, a visible-light-promoted fluorination of an sp^3^-hybridized C-H bond was realized (Scheme 1). The method was optimized for benzylic protons on phenylalanine-like residues and was shown to be efficient even for functionalizing short peptides such as **17** [84].

### 2.2. Elaboration of Side Chain-Fluorinated Amino Acids into Peptides and Proteins

**Scheme 2:**
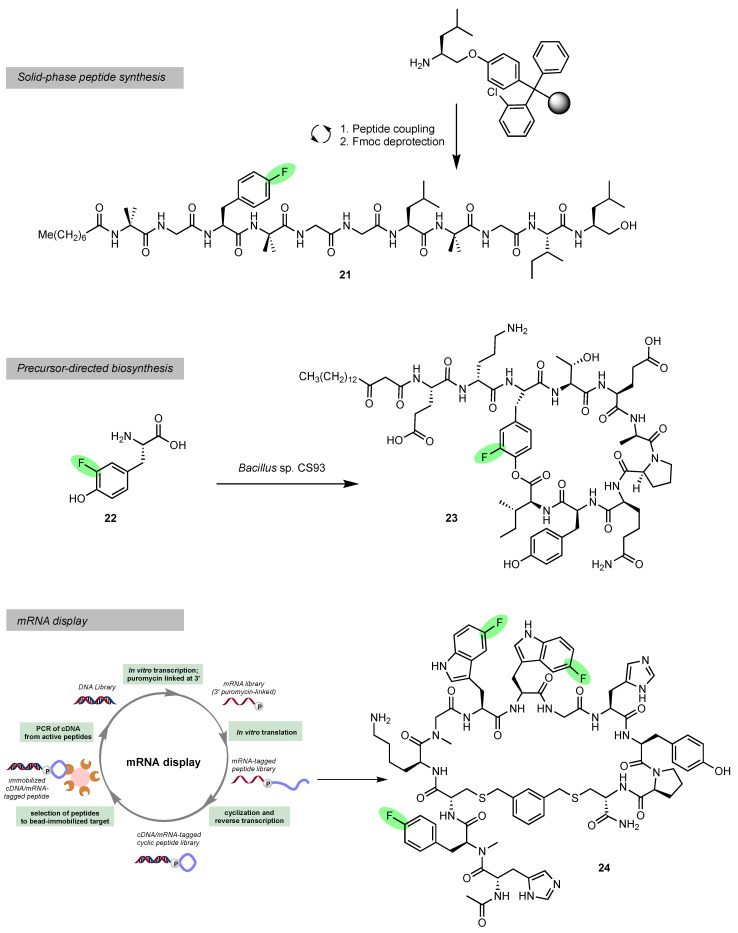
Approaches for elaborating fluorinated amino acids into peptides and proteins.

*Solid-phase peptide synthesis* (SPPS) is one of the most commonly used methods to obtain synthetic peptides containing a side chain-fluorinated residue [85,86,87,88,89,90,91,92,93]. This technique relies on the use of a solid support or resin onto which the desired peptide is assembled. The synthesis is typically performed by initially attaching the *C*-terminal amino acid to the resin. The succeeding amino acids from the target sequence are then individually attached to this anchored residue in a stepwise manner using an appropriate coupling reagent. For instance, SPPS using a leucinol (Lol)-substituted trityl-chloride resin was utilized in introducing (4-fluorophenyl)alanine into an analog of the lipopeptaibiotic trichogin GA IV (**21**, Scheme 2) in order to understand its conformation through ^19^F-NMR studies [88].

In addition to chemical approaches, several biosynthetic pathways for amino acid incorporation into peptides and proteins are available. *Precursor-directed biosynthesis* operates by administering the fluorinated amino acid to a culture of the organism that produces the desired peptide or protein [94,95]. For example, iturins and fengycins are lipopeptides naturally produced by *Bacillus* sp. CS93, which has been shown to exhibit antifungal properties. Feeding the bacterial cultures with fluorinated tyrosine (**22**) resulted in the production of novel fluorinated counterparts of the lipopeptides (e.g., **23**, Scheme 2) [95].

In some cases, biosynthetic incorporation of a fluorinated amino acid into the proteome is actually the mechanism of action of a therapeutic agent. This approach has been studied as a tool for inhibiting the growth of certain bacterial species [96,97,98]. In in vitro cases, fluorinated amino acids fed into bacterial cultures were found to have become misincorporated into the bacterial proteome, ultimately inducing toxicity via inhibition of cell growth [97,98]. A study on the toxicity of *p*-fluorophenylalanine (p-FPA) on *Escherichia coli* 15_T_ revealed that when administered to the culture simultaneously with thymine, p-FPA induced thymine starvation and led to decreased RNA/DNA synthesis, leading to cell cycle arrest [99].

We return now to the situation where a high synthetic yield of a particular fluorinated protein is desired. The rate of incorporation of the fluorinated amino acid can be enhanced by making use of auxotrophic strains that are unable to synthesize a specific amino acid. Typically, the growth medium for the auxotrophic strain is deprived of that amino acid and replaced by a surrogate fluorinated analog, resulting in the incorporation of the latter into synthesized proteins in lieu of its natural amino acid counterpart [100,101,102]. This approach was used in the synthesis of a mutant basic leucine zipper (*bzip*) peptide [102]. Using an auxotrophic *E. coli* strain BL21(DE3), 5,5,5-trifluorolecine and 4,4,4-trifluorovaline were successfully incorporated as isoleucine surrogates.

The amber stop codon (UAG) can be exploited for a similar purpose. This nonsense codon can be included at a specific position of the mRNA where a desired fluorinated amino acid is to be introduced and can therefore be used for site-specific fluorination of a peptide [103,104,105,106]. Using a suppressor tRNA/aminoacyl-tRNA synthetase pair (tRNA^Pyl^_CUA_/MmFAcKRS1) derived from *Methanosarcina mazei*, N^ε^-fluoroacetyllysine (FAcK) was successfully incorporated into the Z_spa_ Affibody (Afb) protein at the position of the amber codon [106].

Finally, display technologies have been explored for the elaboration of side chain-fluorinated amino acids into peptides. One such technique is *mRNA display*, a tool for directed evolution wherein peptides with a desired trait are generated through iterative cycles of diversification and selection (Scheme 2) [107,108,109,110]. The cycle is initiated by transcription of a DNA library to the corresponding mRNA, followed by ligation to puromycin at the 3′ end. Translation of the puromycin-linked mRNA produces mRNA-tagged peptides, which undergo cyclization and reverse transcription into corresponding peptide-mRNA-cDNA fusions. The desired fusions are then selected by binding to a bead-immobilized target. The cDNA from selected fusions subsequently undergoes error-prone PCR to promote amplification and generate the library for the next selection cycle [111,112]. It is possible for the DNA library to be expanded to accommodate unnatural amino acids such as side chain-fluorinated amino acids [111,113]. This approach led to the discovery of cyclic peptide **24** (Scheme 2), containing three side chain-fluorinated amino acid residues for inhibition of proprotein convertase subtilisin-like/kexin type 9 (PCSK 9), which is a valuable target for the treatment of coronary heart disease [107].

## 3. Fluorine, the “Spy”: Transmitting Intelligence on the Properties of Amino Acids, Peptides, and Proteins

Sometimes, when fluorine is introduced into an amino acid side chain, it does not dramatically alter the molecular properties. In such cases, the fluorine might be viewed as an “innocent bystander” or perhaps as a “spy”: it offers the opportunity to gather intelligence about the molecule’s properties through analytical techniques such as ^19^F NMR spectrometry (Section 3.1) or positron emission tomography (Section 3.2). 

### 3.1. ^19^F-Containing Amino Acids as NMR Tags

The introduction of fluorine within a protein provides the opportunity to interrogate the properties and functions of the biomacromolecule through ^19^F NMR spectrometry. This is a longstanding concept that has been the subject of several recent reviews [114,115,116,117,118]. The process begins with the synthesis of a non-natural analog of the protein in which one or more residues are replaced with a side chain-fluorinated amino acid. Next, the ^19^F NMR spectrum of this non-natural protein is recorded as a point of reference, assuming that the presence of fluorine does not dramatically alter the structure compared to the native protein. Starting from this reference point, any subsequent changes in the ^19^F NMR spectrum of the protein can be used to detect, e.g., a conformational change in the protein, a ligand binding event, or some other supramolecular interaction of the protein. 

There are several reasons why fluorine is especially advantageous as an NMR tag. The ^19^F nucleus has 100% isotopic abundance and high NMR sensitivity (83% compared to ^1^H). Since fluorine is not naturally present in any biomacromolecules, there are no background signals even if the protein of interest is present within a complex biological milieu. Finally, ^19^F NMR signals can appear over a very wide chemical shift range (>500 ppm) and they are extremely sensitive to their environment. Taken together, these two features mean that signal overlap is rare even if multiple copies of the same fluorinated amino acid residue are present at different positions in the protein sequence. They also mean that even very subtle changes to a protein’s structure can cause easily detectable perturbations to the ^19^F NMR spectrum. 

**Figure 2:**
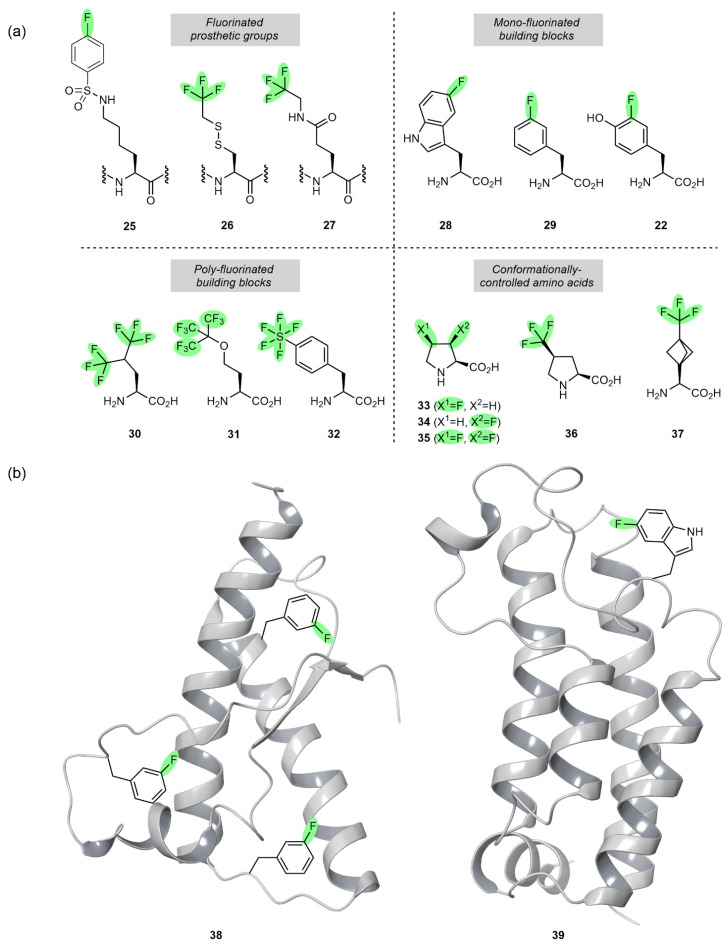
(**a**) Selected examples of fluorinated NMR tags; (**b**) ^19^F NMR spectrometry can be used to interrogate the structure, the conformational dynamics, or the binding events of proteins (e.g., mammalian prion protein, **38**; bromodomain and plant homeodomain-containing transcription factor, **39**).

One way to incorporate fluorine as an NMR tag into a protein is to employ a “prosthetic group” approach [119,120,121,122]. Reagents such as *p*-fluorobenzenesulfonyl chloride or 2,2,2,-trifluoroethanethiol can undergo reaction with solvent-exposed lysine or cysteine side chains, respectively, within an intact protein, delivering a fluorine-labelled structure (e.g., **25**–**26**, Figure 2a). In a more elaborate example, the enzyme transglutaminase was recently shown to accept 2,2,2,-trifluoroethylamine as a substrate, which allowed it to be used as a bioconjugation reagent for the labelling of a glutamate residue on the surface of a model protein (**27**) [123].

A complementary set of NMR tags include the monofluorinated amino acids **28**–**29** and **22** (Figure 2a). Such structures can be considered to be more sophisticated than the prosthetic group examples in the sense that they more closely mimic natural amino acid side chains [88,92,124,125,126,127,128,129,130,131,132,133] and can be installed anywhere in the protein sequence (see Section 2.2), not just at solvent-exposed regions. However, a limitation of **28**–**29** and **22** is that they contain just one fluorine atom, which limits the NMR sensitivity. More recently, polyfluorinated amino acids such as **30**–**32** (Figure 2a) have attracted interest because their intense ^19^F NMR signals potentially allow lower concentrations of the protein analyte to be employed [134].

Fluorinated prolines (e.g., **33**–**36**, Figure 2a) are an interesting subset of NMR tags because they illustrate the importance of identifying a conformational match with the native amino acid [89,116,135,136]. 4-Fluoroprolines (e.g., **33**) and 3-fluoroprolines (e.g., **34**) adopt different puckers of the 5-membered ring (see also Section 5.1) and different *cis*/*trans* ratios of the peptide bond to the *N*-terminal side of the proline residue. In these cases, the fluorine is no longer an “innocent bystander”. However, if two fluorine substituents are installed at the 3- and 4-positions, with appropriate stereochemistry (e.g., **35**), then the conformational influences of the two fluorines offset one another, resulting in a fluorinated analog that has very similar conformational characteristics to natural proline [137]. Another fluorinated proline derivative that has been found to closely mimic the conformational characteristics of proline itself, while providing an intense ^19^F NMR signal, is the trifluoromethyl-containing analog **37** (Figure 2) [89].

Having seen some examples of amino acids that bear ^19^F NMR labels (Figure 2a), let us turn our attention to the application of these building blocks and the study of protein structure and function. 

The conformational dynamics of proteins, including their folding and unfolding processes, are fundamental aspects of biology [118,130]. A medicinally relevant case is the misfolding of proteins into amyloid fibrils, which is the basis of diseases such as Alzheimer’s disease and Creuzfeldt–Jakob disease. Mammalian prion protein (PrP) is a predominantly α-helical protein that is associated with neuronal cell membranes. Under certain circumstances, PrP can misfold into a β-sheet rich structure. The misfolded structure catalyzes the misfolding of further molecules of PrP, and this autocatalytic process leads to the accumulation of aggregates called amyloid fibrils, which can cause neuronal cell death and disease pathology. To study the pathway of amyloid formation, a protein-observed ^19^F NMR study was undertaken [138]. Three 3-fluorophenylalanine (**29**) residues were introduced into the protein to replace the Phe141, Phe175, and Phe198 of PrP (**38**, Figure 2b). The ^19^F NMR spectrum of the fluorinated protein (**38**) revealed the presence of several oligomeric species, with the predominant constituent being assigned as an octamer. Variable-temperature ^19^F NMR experiments then allowed the equilibrium distributions to be perturbed and the thermodynamic driving forces of aggregation to be elucidated. The protein-observed ^19^F NMR approach was also able to explain how certain mutations make the PrP protein more prone to aggregation through the stabilization of the octamer [138].

Another application of ^19^F-labelled proteins is to discover and optimize small molecule ligands. An example of this approach is seen with the protein known as BPTF, or “bromodomain and plant homeodomain-containing transcription factor”. This protein is involved in the regulation of chromatin accessibility, and its overexpression is associated with lung cancer. A 5-fluorotryptophan residue (**28**) was incorporated at the binding surface of BPTF (**39**, Figure 2b), which enabled a medium-throughput screen of ~200 potential ligands to be performed, using ^19^F NMR spectrometry as the detection technique [117]. This screen resulted in a promising hit molecule, the subsequent structure-activity optimization of which was also facilitated by protein-observed ^19^F NMR.

Protein-observed ^19^F NMR can be used to study the interactions of proteins with other biomacromolecular structures such as membranes. When investigating the membrane interactions of helical antimicrobial peptides by solid-state NMR, it is helpful to have a fluorine tag that is held in a fixed orientation relative to the helical axis [85,139,140,141,142,143]. The unusual amino acid **37** (Figure 2a), which contains a rigid bicyclo[1.1.1]pentane moiety, meets this requirement [144,145].

^19^F NMR spectrometry can be applied not only to interrogate the properties of a fluorinated molecule as described above, but alternatively to visualize where a fluorinated molecule travels within the body (i.e., ^19^F magnetic resonance imaging, or ^19^F-MRI). For example, 6-fluoro-DOPA, a ring-fluorinated analog of dihydroxyphenylalanine, has been employed as a brain imaging agent in a rat model of Parkinson’s disease [146]. A multi-fluorinated DOPA analog has also been developed in order to achieve a stronger signal for ^19^F-MRI applications [147]. However, the use of ^19^F NMR spectrometry as an imaging modality remains quite niche [148], particularly in comparison with PET, which is discussed in the next section.

### 3.2. ^18^F-Labelled Amino Acids and Peptides as PET Tracers

^18^F-Radiolabelled amino acids can be valuable agents for the diagnosis and visualization of cancer. Tumour cells have high biosynthetic demand [149], and one way that they can secure an increased supply of biosynthetic building blocks is by upregulating amino acid transporter proteins [150,151]. Thus, ^18^F-radiolabelled amino acids often selectively accumulate in tumours, where their presence can be detected by positron emission tomography. 

**Figure 3:**
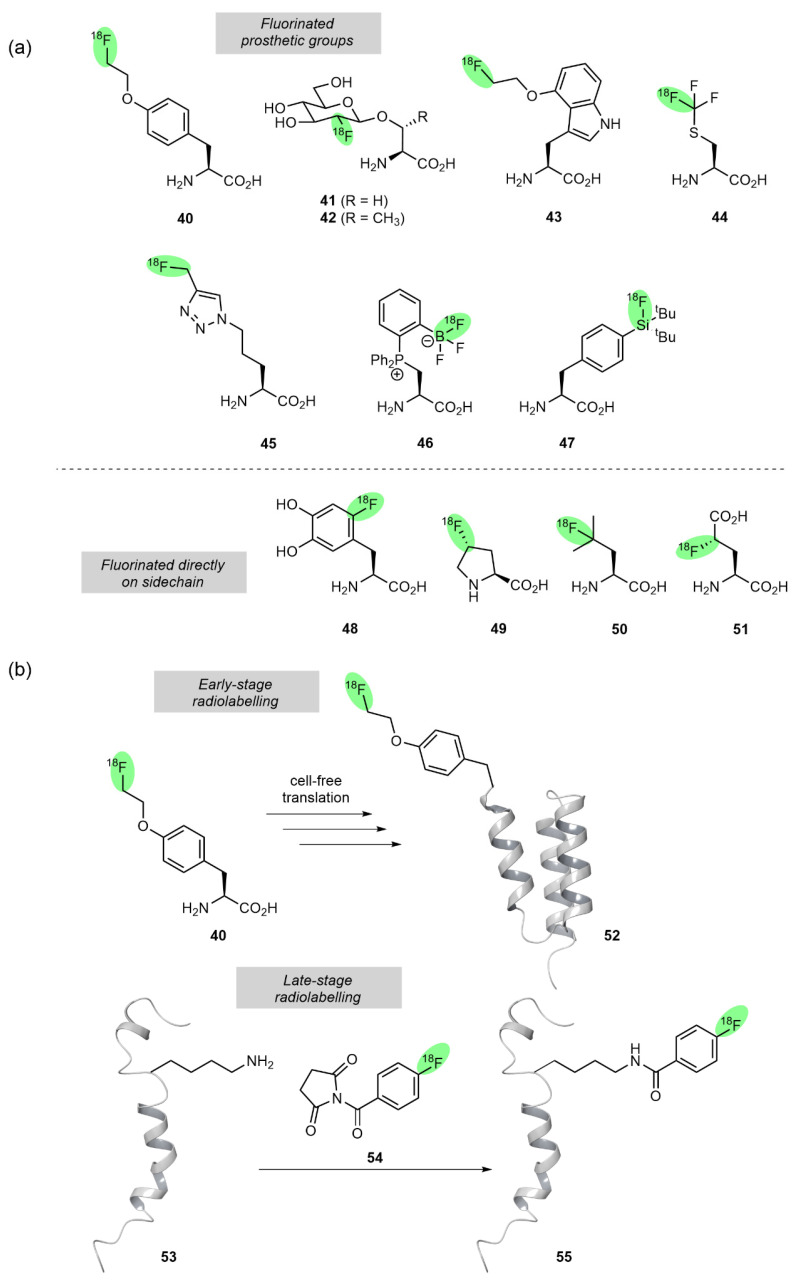
(**a**) Amino acids with ^18^F-radiolabelled side chains; (**b**) early- vs. late-stage radiofluorination of proteins (human epidermal growth factor 2, **52**; parathyroid hormone, **55**).

One of the most operationally straightforward ways to incorporate an ^18^F radiolabel onto the side chain of an amino acid is to install a prosthetic group that contains ^18^F (Figure 3a). For example, the tyrosine side chain contains a phenol moiety, which can be alkylated with [^18^F]fluoroethyltosylate to provide 2-[^18^F]fluoroethyltyrosine (**40**). This important radiotracer is particularly valuable for the imaging of brain cancers as the amino acid transporter proteins mentioned above enable this tracer to efficiently cross the blood–brain barrier [150,152,153,154]. Prosthetic groups for the radiolabelling of several other amino acids besides tyrosine [155,156,157] have also been developed, taking advantage of the reactivity of the side chains of serine (**41**) [158], threonine (**42**) [158], tryptophan (**43**) [159], cysteine (**44**) [160,161], and ornithine (**45**) [151,162] (Figure 3a). Certain more elaborate prosthetic groups afford the alternative possibility of late-stage fluorination through ^18^F/^19^F isotopic exchange (e.g., **46**–**47**) [163,164].

A potential disadvantage of the prosthetic group approach is that the structure of the labelled amino acid has become rather different from the natural amino acid, so the in vivo distribution might also differ. To overcome this issue, it is sometimes desirable to attach the ^18^F atom directly to the amino acid side chain as a replacement for a C–H hydrogen (Figure 3a). Most commonly, a synthetic precursor bearing a leaving group is required to enable an S_N_Ar [65,165,166,167,168,169,170] or S_N_2 reaction [171,172,173,174,175,176,177,178,179,180,181] to take place to install the ^18^F substituent (e.g., **48**–**49**). Some other reaction manifolds have also been exploited for radiofluorination of amino acid side chains or precursors thereof, including direct C–H fluorination (**50**) [182,183,184] and organocatalytic electrophilic fluorination (**51**) [76].

More complex structures have also been created in which the ^18^F-radiolabel is attached to an amino acid side chain within a peptide or protein architecture. This opens up the potential to visualize a variety of different disease states, depending on the biomacromolecule that is radiolabelled. There are broadly two strategies for synthesizing a peptide or protein in which one amino acid side chain bears an ^18^F-radiolabel: early-stage vs. late-stage fluorination.

The early-stage radiofluorination strategy commences with an ^18^F-labelled amino acid and then elaborates it into a peptide or protein (Figure 3b). Examples of this strategy are quite rare due to the challenge of synthesizing an entire peptide or protein on a short timescale, but the challenge can be met by leveraging biosynthetic machinery for the elaboration task. For example, 2-[^18^F]fluoroethyl tyrosine (**40**) was elaborated via a cell-free translation system into a small protein (or “affibody”) that binds to the human epidermal growth factor receptor (**52**, Figure 3b) [185]. This biosynthesis afforded a 6.5% overall radiochemical yield.

The second strategy for ^18^F-radiolabelling a peptide or protein is to perform a late-stage derivatization of one amino acid side chain within the biomacromolecule (Figure 3b). Lysine and cysteine are the most commonly targeted amino acids for this purpose [86,90,91,186,187,188,189,190,191]. For example, a lysine side chain within the 34-residue parathyroid hormone (**53**) was derivatized as the *p*-[^18^F]fluorobenzoyl amide, generating a macromolecular radiotracer (**55**) suitable for the study of osteoporosis [189]. In another example, a cysteine side chain within the 36 kDa protein annexin V was derivatized with ^18^F via a maleimide adduct, generating a macromolecular radiotracer capable of detecting apoptotic cells [190].

## 4. Fluorine, the “Tinker”: Improving the Pharmacokinetic Properties of Amino Acids, Peptides, and Proteins

The sub-optimal pharmacokinetic properties of peptides are one of the key obstacles to their development into viable drugs [15,17]. There is some evidence that fluorination of amino acid side chains can help to improve the hydrophobicity, permeability, and/or stability of the metabolism of certain amino acids and peptides [192]. Selected examples are presented below.

### 4.1. Hydrophobicity and Permeability

**Figure 4:**
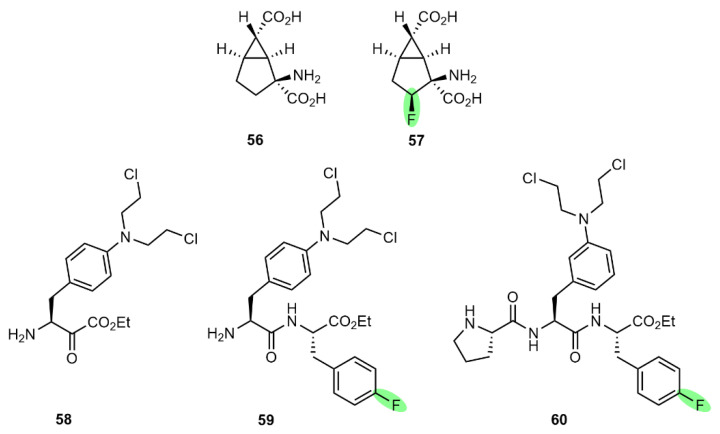
The oral bioavailability of amino acid-based medicinal agents **56** and **58** is enhanced in the side chain-fluorinated analogs **57**, **59**, and **60**.

The bicyclic amino acid **56** (Figure 4) is a potent agonist of the metabotropic glutamate receptor, and it shows promise for the treatment of a variety of central nervous system disorders including schizophrenia. Compound **56** suffers from poor oral bioavailability, but this limitation is impressively overcome in the fluorinated analog **57**, which replicates the agonist activity of **56** in vitro while being far more efficacious in vivo (Figure 4) [193]. It is unclear whether the improved oral bioavailability of **57** is attributable to increased hydrophobicity, to greater resistance to metabolism, or to some other effect.

Mephalan (**57**, Figure 4) is a DNA-targeted anticancer drug featuring a nitrogen mustard moiety located on the side chain of phenylalanine. A major limitation of compound **58** is its inability to traverse cell membranes. This limitation can be overcome through a prodrug approach, in which mephalan is temporarily masked as a di- or a tripeptide (e.g., **59**–**60**, Figure 4) [194,195]. The presence of the fluorinated amino acid in **59**–**60** enhances the drugs’ membrane permeability, and thereby substantially boosts the anticancer potency in each case. 

### 4.2. Stability towards Proteolysis

When it comes to the issue of proteolytic stability, much work has been done in terms of fluorination of the peptide *backbone* (e.g., peptidomimetics containing fluoroalkenes or –C(CF_3_)=CH– groups as non-hydrolyzable isosteres of the amide bond). By contrast, fluorinating peptide *side chains* is a less intuitive strategy for imparting proteolytic stability because in such structures the potentially hydrolyzable amide functional group is retained; essentially the hope is that a fluorinated side chain would be incompatible with the corresponding binding pocket within a protease enzyme’s active site, preventing its hydrolytic action. There is some evidence that this strategy can indeed impart proteolytic stability to peptides, but only in particular cases, not as a general trend [196,197,198,199].

**Figure 5:**
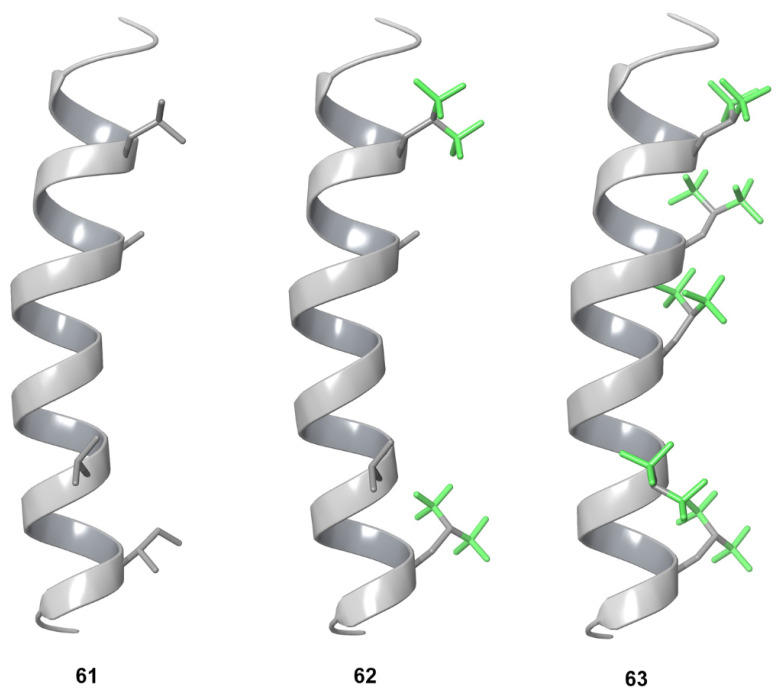
Magainin (**61**) is highly susceptible to proteolysis. The fluorinated analogs **62**–**63** (containing two and five hexafluoroleucine residues, respectively) are progressively more resistant to proteolysis.

The α-helical peptide magainin (**61**, Figure 5) exhibits antimicrobial activity through its ability to assemble into toroidal pores within the bacterial cell membrane. However, **61** contains several trypsin cleavage sites, which leads to a short half-life in vivo and limits the usefulness of **61** as a pharmaceutical agent. The fluorinated analogs **62** and **63** (Figure 5), which contain two or five hexafluoroleucine residues positioned along the hydrophobic face of the helix, respectively, show either a modest or a dramatic increase in proteolytic stability, due to steric incompatibility of the fluorinated side chains with the protease active site. However, the increased proteolytic stability of **63** comes at a price: this fluorinated peptide has lower antimicrobial activity than **61**, due to its propensity to self-assemble into helical bundles in aqueous solution rather than toroidal pores within the bacterial membrane (the aggregation behavior of other highly fluorinated peptide helices is discussed in Section 5.4).

A related approach that can deliver increased proteolytic stability is to incorporate a fluorinated side chain as an *additional* structural feature. α,α-Disubstituted amino acids, in which a trifluoromethyl side chain is present in addition to a canonical side chain, can endow peptides with greater proteolytic stability if the location and stereochemistry of the trifluoromethyl substituent causes a clash within the protease active site [200].

In contrast with the *aliphatic* fluorinated side chains such as those seen in **62**–**63** (Figure 5), the presence of *aromatic* fluorinated side chains seldom leads to greater proteolytic stability. For example, *p*-fluorophenylalanine has been incorporated into a variety of short peptides and globular proteins as a replacement for natural phenylalanine, but this usually leads to greater susceptibility, not resistance, to protease digestion [131,199,201,202,203,204,205,206]. This can be attributed to the likely ability of the *p*-fluorophenylalanine side chain to also bind efficiently within protease binding pockets that have evolved to accommodate natural phenylalanine. Indeed, the design of the prodrugs **59**–**60** (Figure 4) highlights cases in which efficient hydrolysis of an amide bond adjacent to *p*-fluorophenylalanine is a desirable event as part of the prodrug strategy.

### 4.3. Resistance to P450 Oxidation

**Figure 6:**
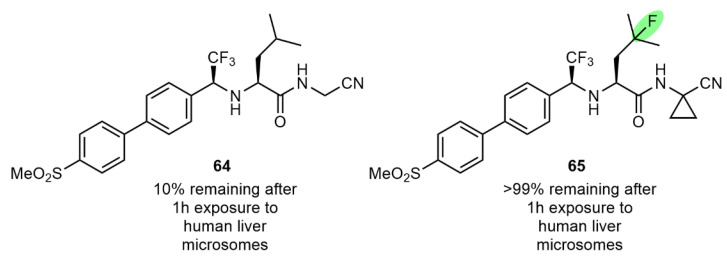
Fluorination of an amino acid side chain confers resistance to P450 metabolism.

Compound **64** (Figure 6) is a potent inhibitor of the protease enzyme cathepsin K. As such, it is a promising lead compound for the treatment of osteoporosis. Compound **64** suffers from rapid metabolism in the body, due to the action of a cytochrome P450 enzyme (CYP3A), which catalyzes the hydroxylation of the leucine side chain of **64**. This metabolic process is prevented in the fluorinated next-generation analog **65** (Figure 6), leading to dramatically enhanced bioavailability [207].

## 5. Fluorine, the “Tailor”: Folding Amino Acids, Peptides, and Proteins into Precise 3D Shapes 

In the world of amino acids, peptides, and proteins, conformation is inextricably linked to function. Therefore, methods for controlling conformation can have a variety of valuable applications. Conformational control can be considered across a range of scales, from the individual amino acid level (Section 5.1) [73], to the peptide secondary structure (Section 5.2), to the protein tertiary structure (Section 5.3), and even to the quaternary structure (Section 5.4). Fluorination can have significant impacts on all of these scales.

### 5.1. Conformational Control at the Individual Amino Acid Level

Fluorine offers a unique ability to control the conformations of individual amino acid side chains. The polar C–F bond tends to align in predictable ways with neighboring functional groups [208,209]. For example, molecules containing a N^+^–C–C–F moiety preferentially adopt conformations in which the N^+^ and F^δ−^ atoms are *gauche*, due to electrostatic attraction. In another example, α-fluoroamides (i.e., molecules containing a F–C–C(=O)–NH moiety) preferentially adopt a conformation in which the CF and CO bonds are *anti*-periplanar, which can be rationalized in terms of dipolar forces. In yet another example, molecules containing a F–C–C–H moiety preferentially adopt a conformation in which the CF and CH bonds are *anti*-periplanar, due to σ_CH_→σ*_CF_ hyperconjugation (note that this latter effect can reinforce the conformational preference described above for N^+^–C–C–F compounds). 

**Figure 7:**
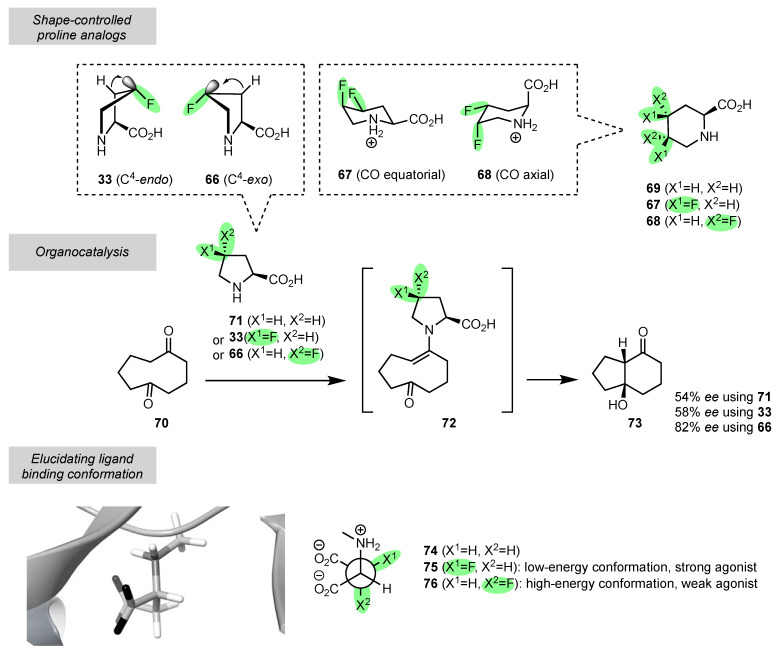
Controlling the conformations of individual amino acid side chains.

Such conformational effects can be exploited to control the conformations of amino acid side chains, and this has been demonstrated most notably for proline [27,210,211,212,213,214,215,216]. For example, fluorination at the 4-position of the proline side chain (i.e., **33** and **66**, Figure 7) can stabilize either the C^4^-*endo* or C^4^-*exo* pucker depending on the configuration of the fluorinated stereocentre, which is attributable to σ_CH_→σ*_CF_ hyperconjugation in each case. This ability to control the pucker of the proline ring has been exploited for a range of applications, including enhancing the enantioselectivity of organocatalytic reactions (e.g., **70**→**73**, Figure 7) [217,218,219]. Fluorination at the 3-position of the proline side chain can influence the pucker in a similar way [220,221].

Fluorine can also influence the conformation of the ring-expanded proline analog, pipecolic acid (**69**, Figure 7) [222,223]. The six-membered ring of **69** preferentially adopts a chair conformation in which the carboxyl group is equatorial. This conformation is maintained in the difluorinated analog **67** (Figure 7), with the N^+^–C–C–F and F–C–C–F moieties both adopting favorable *gauche* alignments. In contrast, the diastereoisomeric analog **68** adopts a ring-flipped conformation in which the carboxyl group is forced into the axial position. 

The ability of fluorine to control the conformations of proline analogs will be further examined in Section 5.2, Section 5.3 and Section 6.2.

Another context in which fluorine-derived conformational control of amino acid side chains can be valuable is in the elucidation of the binding conformation of certain receptor ligands. For example, the *N*-methyl-d-aspartate (NMDA) receptor is a target of interest for the treatment of several disorders of the nervous system. An X-ray crystal structure of this receptor bound to its natural ligand (NMDA, **74**) reveals a “bent” ligand conformation in which the carboxylate substituents of **74** are *gauche* to one another (Figure 7) [224]. This information is supported by the relative activity of two fluorinated NMDA analogs, **75** and **76** (Figure 7) [225]. For analog **75**, binding leads to a favorable *gauche* N^+^–C–C–F alignment, and as a result, this ligand exhibits strong agonism almost equal to that of the native ligand, **74**. In contrast, for analog **76**, binding would require an unfavorable *anti*-N^+^–C–C–F angle, and as a result, this ligand is virtually inactive. 

### 5.2. Peptide Secondary Structure 

We have seen that fluorine can influence the conformations of individual amino acid side chains (Section 5.1). Now, if a fluorinated amino acid is elaborated into a peptide, the fluorine-derived conformational control can sometimes extend beyond the individual amino acid and can also start to influence the preferred rotameric species along the peptide backbone. 

**Figure 8:**
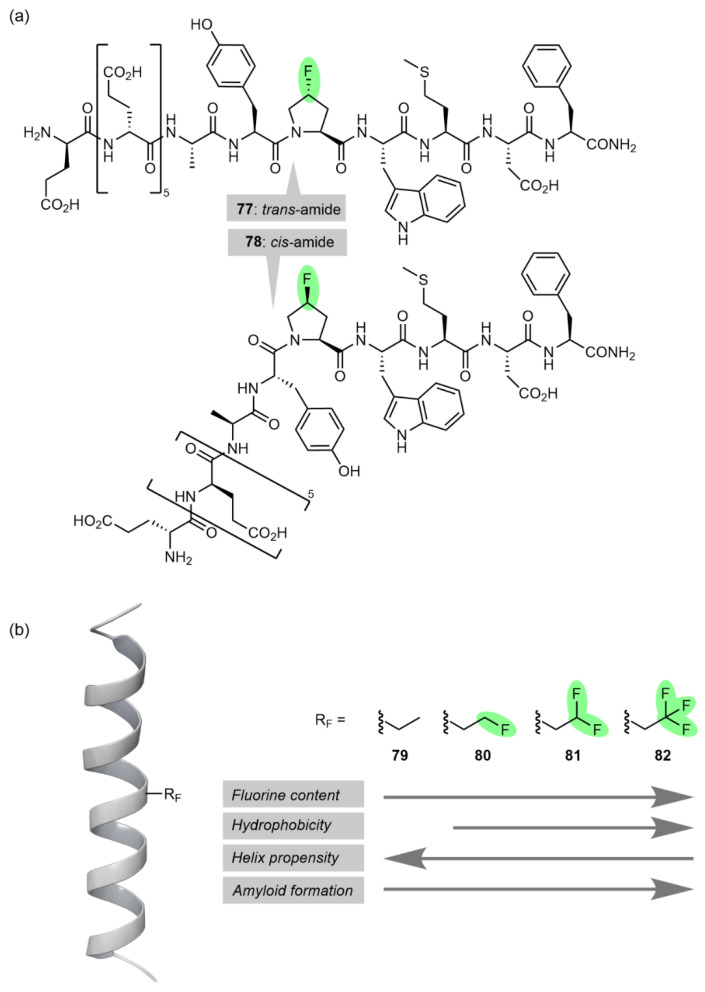
Influencing peptide secondary structure through side chain fluorination: (**a**) hairpin turn formation; (**b**) helix disruption.

Consider again the example of fluorinated proline. It has been established that in peptides containing *trans*-4-fluoroproline (e.g., **77**, Figure 8a), the amide bond preceding the fluoroproline residue strongly favors the *trans*-conformation [93,226,227,228]. In contrast, peptides containing *cis*-4-fluoroproline (e.g., **78**, Figure 8a) favor a *cis*-amide conformation adjacent to the fluoroproline residue. This contrast can be exploited to alter the target-binding properties of a bioactive peptide. For example, the peptide sequence in **77**–**78** is derived from the gastrin hormone G17. This hormone binds to a G-protein coupled receptor called cholecystokinin-2 (CCK-2R), which is overexpressed in a range of cancers. The key binding motif of G17 is thought to adopt a compact, hairpin-like structure; this conformation is better replicated by the *cis*-4-fluoroproline-containing peptide **78** (Figure 8a), endowing this peptide with higher CCK-2R binding affinity than the *trans*-4-fluoroproline-containing peptide **77** [93].

The idea of fluorine-derived conformational control holding small peptides into desired shapes for binding to targets is further examined in Section 6.3.

The ability of side chain-fluorinated amino acids to influence peptide secondary structure is not just limited to hairpin turns: α-helices and β-sheets can be affected too [229]. For example, peptide **79** (Figure 8b) is an engineered structure that is capable of adopting either an α-helix or a β-sheet conformation. When fluorine atoms are successively introduced into one side chain, the helix is destabilized to a greater and greater extent [230]. This can be attributed to the increased hydrophobicity of the highly fluorinated side chains, which are not favorably accommodated at the water-exposed edge of the helix. Intriguingly, in this peptide scaffold, there is an inverse relationship between α-helical and β-sheet propensity. This knowledge about the effects of side chain hydrophobicity on the kinetics and thermodynamics of β-sheet formation could be relevant in the future for designing amyloid-based materials, or perhaps even in understanding the progression of amyloid-based diseases. 

It should be noted that the outcome can be different if not one, but *multiple* fluorinated residues are incorporated along the same edge of an α-helix [231]. In some cases, the helix can be stabilized because it contains a “fluorous edge”, which can engage in favorable supramolecular aggregation phenomena. This concept was mentioned in Section 4.2 and is explored further in Section 5.4 of this review. 

### 5.3. Protein Tertiary Structure

**Figure 9:**
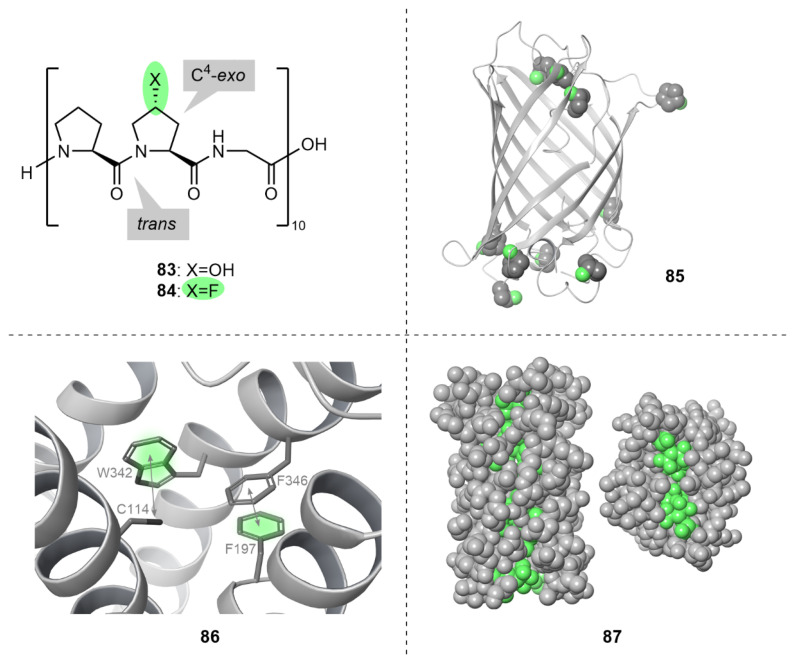
Influencing protein tertiary structure through side chain fluorination.

Side chain-fluorinated amino acids can act as protein “superfolders”. For example, collagen, which consists of many tripeptide repeats of typical structure **83** (Figure 9), must adopt an all-*trans* conformation in order to assemble into its final triple-helical structure. Fluorine can accelerate this folding process. The fluorine substituent in the non-natural collagen analog **84** exerts an inductive pull that lowers the amide C–N bond order, reducing the kinetic barrier to *cis*/*trans* isomerization, and thereby helping the peptide chain to find its way to the required all-*trans* conformation [232]. The fluorine also provides thermodynamic stabilization of the final structure by favoring the C^4^-*exo* pucker, which matches the pucker found in natural collagen [233].

Fluoroprolines have been incorporated into several other proteins besides collagen [211,212,214,215,234,235,236,237]. For example, fluoroproline incorporation has led to a “superfolding” analog of green fluorescent protein (GFP). The crystal structure of GFP reveals that the majority of its proline residues (9 out of 10) adopt the C^4^-*endo* pucker [238]. An analog of GFP in which all 10 proline residues are replaced with *cis*-4-fluoroproline (**85**, Figure 9) displays enhanced folding characteristics, attributable to (i) lowering of the kinetic barrier to *cis*/*trans* peptide bond isomerization, as described above for collagen, and (ii) thermodynamic stabilization of the required C^4^-*endo* pucker [239]. In contrast, the corresponding GFP analog containing *trans*-4-fluoroprolines does not fold efficiently and lacks fluorescent properties. 

Fluorination can modulate protein tertiary structures in other ways too. For example, fluorination can be exploited to enhance or disrupt a specific interaction within a protein core, in order to assess the contribution of that interaction to the protein’s structure and/or function [58,139,240,241]. This idea has been applied to study both aromatic–aromatic and sulfur–π interactions within the dopamine D_2_ receptor (**86**, Figure 9) [242]. Analogs of **86** were created in which fluorine atoms were successively incorporated, separately, into the side chains of Phe197 and Trp342. Increasing levels of fluorination were found to correlate with reduced protein function in both cases, and this was taken as evidence that Phe197 and Trp342 probably participate as electron-rich components within the native protein in aromatic–aromatic and sulfur–π interactions, respectively. Cation–π interactions within other proteins have also been interrogated in a similar fashion [242,243,244,245].

Having seen examples of individual, targeted interactions mediated by fluorine within a protein core, let us now consider a scenario where there are *multiple*, *non-specific* interactions. Proteins have been engineered to have multiple, highly fluorinated amino acids buried within the hydrophobic interior (e.g., **87**, Figure 9) [246,247,248,249,250,251]. For example, protein **87** contains 12 hexafluoroleucine residues (i.e., 72 fluorine atoms) whose side chains form a highly fluorinated spine within the protein core. Such “fluorous core” proteins typically display markedly greater structural stability compared with their non-fluorinated counterparts (e.g., ΔG°_fold_ = −27.6 kcal/mol for **87**, compared with ΔG°_fold_ = −18.0 kcal/mol for the non-fluorinated parent protein) [251]. The increased structural stability of “fluorous core” proteins such as **87** can be rationalized by comparing the relative energies of the unfolded and folded states [87,137,251,252,253]. In the unfolded state, the perfluorinated side chains of **87** are solvent-exposed; this is unfavorable because the fluorinated moieties cannot interact attractively with the water solvent, and also because the fluorinated moieties occlude the backbone NH and CO groups from hydrogen bonding with water. These unfavorable phenomena are avoided in the folded state. 

### 5.4. Protein Quaternary Structure

We consider again the structure of the “fluorous-core” protein **87** (Figure 9). It comprises a bundle of four α-helices, which are connected from one to the next via peptide loops. It is intriguing to consider a hypothetical scenario in which the loops were removed so that **87** was no longer a single protein but rather four separate α-helical peptides. Would the fluorous packing effect be strong enough to induce separate “fluorous-edged” peptide helices to come together and form higher-order structures?

**Figure 10:**
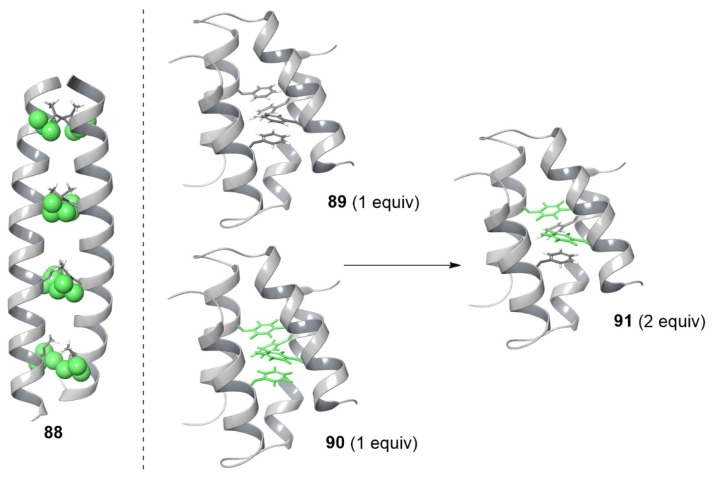
Controlling protein aggregation through side chain fluorination.

The answer is yes [254]. Several peptide systems have been engineered that can self-assemble, zipper-like, into aggregates via a fluorous interface (e.g., **88**, Figure 10) [21,197,198,255,256,257,258,259,260,261,262,263,264]. A notable feature of this fluorine-directed self-assembly phenomenon is that it can be engineered to occur either within *hydrophilic* peptide helices (which can dimerize in aqueous solution) or within *hydrophobic* peptide helices (which can dimerize while embedded within a lipid membrane) [265,266]. This versatility comes from the amphipathic character of the “fluorous edge”.

Self-association phenomena have been investigated with other fluorinated peptide systems too. Certain peptides have a propensity to aggregate into supramolecular architectures like fibrils or hydrogels. Such aggregation-prone peptides can have a wide range of sizes, but a common feature is the presence of phenylalanine residues within the sequence. In several cases, the replacement of phenylalanine residues with fluorophenylalanine has been found to modify the propensity for self-assembly and/or the mechanical properties of the resulting supramolecular architecture [267,268,269,270].

All of the quaternary structures that we have examined so far have been of *homomeric* species. We will now conclude this section with an example of *heteromeric* peptide aggregation that is driven by fluorine-based interactions [271]. The first peptide of interest is a designed 32-residue scaffold known as α_2_D (**89**, Figure 10). In the absence of any other peptides, α_2_D folds and aggregates into a homodimer in which four phenylalanine side chains (two from each monomer) form stacking interactions at the dimer interface. This homodimeric folding pattern is also seen with a fluorinated analog of α_2_D (**90**, Figure 10) in which the phenylalanine residues are replaced with pentafluorophenylalanine. Remarkably, however, if the two homodimers **89**–**90** are mixed together, they re-assemble selectively into heterodimers (**91**) as depicted in Figure 10. This preference for heterodimerization can be explained by the quadrupolar attraction of the phenyl group of **89** with the pentafluorophenyl group of **90**.

## 6. Fluorine, the “Soldier”: Guiding Amino Acids, Peptides, and Proteins to Hit Their Biological Targets 

Our attention now turns from “within” a biomolecule to its surroundings, i.e., how an amino acid or peptide interacts with its broader environment. Fluorination can modulate this in useful ways, for example, in the design of mechanism-based enzyme inhibitors (Section 6.1), in the enhancement of the intermolecular forces between an amino acid side chain and its biological target (Section 6.2), or through conformational pre-organization of a bioactive peptide (Section 6.3). 

### 6.1. Mechanism-Based Enzyme Inhibitors

**Scheme 3:**
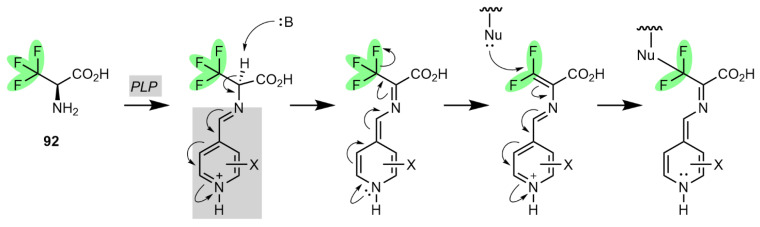
β,β,β-Trifluoroalanine (**92**) is a mechanism-based inhibitor of several pyridoxal phosphate (PLP) dependent enzymes.

Side chain-fluorinated amino acids have proven to be useful as mechanism-based inhibitors of a range of pyridoxal phosphate (PLP) dependent enzymes, including amino acid racemases, decarboxylases, and transaminases [272,273,274]. Several such enzymes are of medicinal importance as targets for the treatment of African sleeping sickness, hirsutism, epilepsy, and cancer [275]. A fluorine atom strategically located at the β-position of a substrate mimic (e.g., **92**, Scheme 3) provides a leaving group that can ultimately lead to irreversible alkylation, and hence inactivation, of the enzyme.

### 6.2. Enhancing Intermolecular Forces

Fluorination can also enhance the *non-covalent* binding of amino acids and peptides towards their biological targets. This is possible because fluorination can modulate intermolecular forces. 

**Figure 11:**
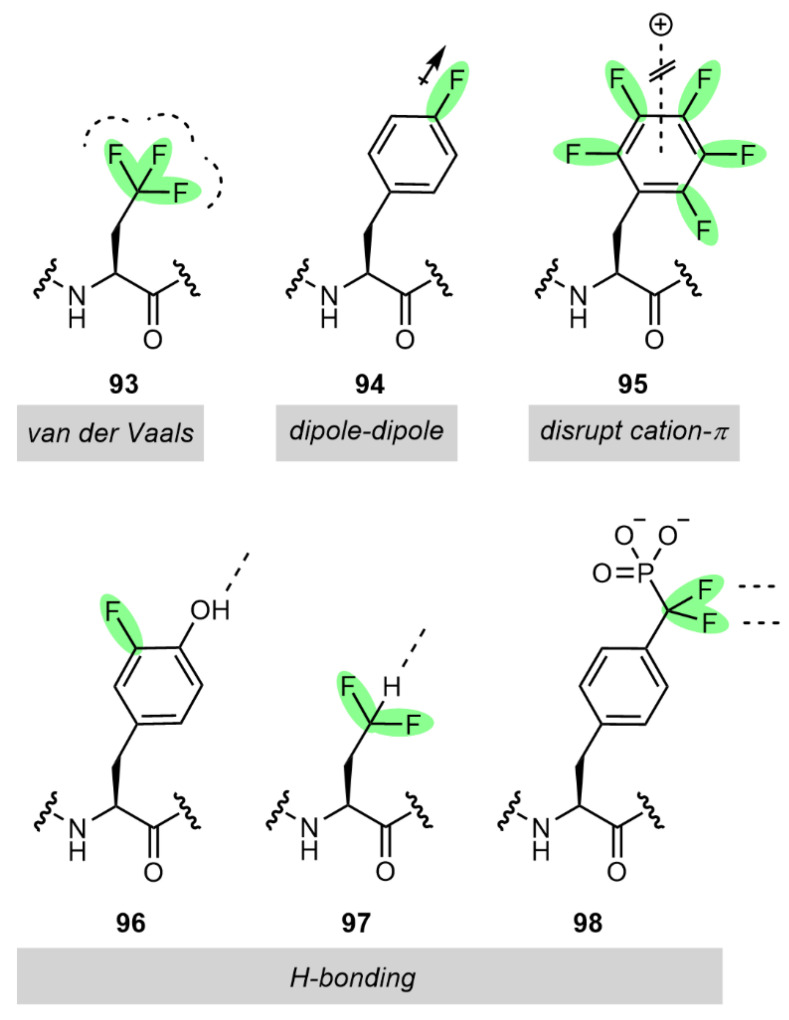
Fluorine can modulate the intermolecular forces that amino acid side chains engage in.

The first type that we will consider is hydrophobic (or van der Vaals) forces [276]. In some instances, the presence of a fluorinated substituent like -CF_3_ on the side chain of a peptide ligand (e.g., **93**, Figure 11) [277] can provide good size- and shape-complementarity within a binding pocket of a biomacromolecule; the fluorines contribute to the binding interaction simply by presenting an appropriately shaped hydrophobic volume. 

In other instances, fluorination can deliver more targeted interactions within a binding site. For example, a common structural motif within peptide-based drugs is a fluorophenylalanine residue (e.g., **94**, Figure 11) [37,278,279,280,281]. The presence of a polar C–F bond within the phenylalanine side chain of **94** offers the opportunity for adventitious dipolar interactions to be achieved within a hydrophobic binding pocket [282], leading to enhanced affinity. This is separate from the other benefits that we have already seen in terms of the pharmacokinetic properties of fluorophenylalanines (see Section 4). Other aromatic amino acids (e.g., tryptophan) have also been fluorinated as a means to alter their dipolar character and enhance their bioactivity [283].

Another type of intermolecular force that aromatic amino acid side chains can participate in is cation–π interactions. As discussed in Section 5.3, multiply-fluorinated aryl side chains can be incorporated into proteins in order to disrupt, and thereby measure the importance of, cation–π interactions to the protein’s tertiary structural integrity [242,243,244,245]. Those examples are technically *intramolecular* in nature. It is also possible to employ fluorination to study *intermolecular* cation–π interactions [284,285,286]. For example, pentafluorophenylalanine residues (i.e., **95**, Figure 11) have been introduced into ion channel proteins in order to interrogate the contribution of cation–π interactions in the binding of ion channel blockers such as tetrodotoxin [287,288].

Fluorine substituents can also alter the hydrogen-bonding character of amino acid side chains. For example, fluorine has been introduced into tyrosine side chains (e.g., **96**, Figure 11) as a means of modulating the hydrogen-bonding ability of the adjacent phenolic group through inductive effects [289,290]. This strategy has been applied to optimize the binding of fluorinated small-molecule ligands to their cognate receptors (e.g., 3-fluorotyrosine binding to an amino acid transporter protein). The conceptual inverse, in which a *protein* is fluorinated in order to alter its interactions with non-fluorinated small-molecule binders, is also possible. For example, the enzyme glutathione *S*-transferase contains a key tyrosine residue within its active site. Replacement of this key residue with 3-fluorotyrosine (**96**) resulted in an altered hydrogen-bonding ability within the active site and correspondingly altered reaction kinetics, which is information that helped to reveal the native enzyme’s catalytic mechanism [291,292].

The difluoromethyl (-CF_2_H) substituent (e.g., **97**, Figure 11) is another example of a fluorinated hydrogen-bonding moiety that has been successfully incorporated into amino acid side chains to enhance target-binding affinity [293,294,295].

Finally, fluorine can be useful for the design of bioisosteres of post-translationally modified amino acids [296]. A common type of post-translational modification is the phosphorylation of tyrosine residues (i.e., to provide Ar-O-PO_3_^2−^). Aryl phosphonates (i.e., residues containing Ar-CH_2_-PO_3_^2−^) are non-hydrolyzable isosteres of tyrosine phosphates, and they can serve as inhibitors of phosphatase enzymes. However, the inhibitory potency is dramatically enhanced with the more advanced isosteres, difluorophosphonates (i.e., Ar-CF_2_-PO_3_^2−^, e.g., **98**, Figure 11) [46,297]. The fluorine substituents provide a closer mimicry of the phosphate group in terms of the hydrogen bond acceptor ability, but also in terms of the p*K*_a2_ and even the Ar-X-P bite angle [209].

### 6.3. Conformational Pre-Organization

Aside from the modulation of intermolecular forces (Figure 11), another way that fluorine can enhance the binding affinity of a peptide ligand towards its target is through conformational control [221]. If a ligand is flexible, then an entropic penalty must be paid upon target binding. However, if the ligand can be pre-organized into the target-binding conformation, then the entropic cost is pre-paid, and this can translate into higher binding affinity. 

**Figure 12:**
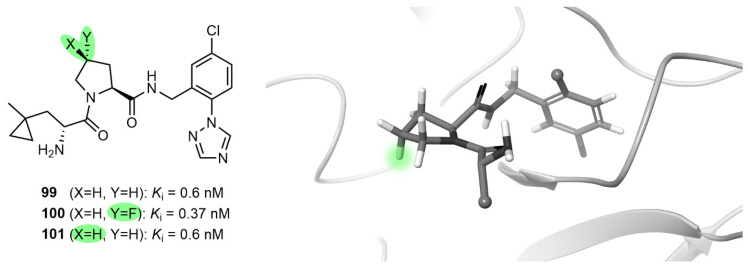
Conformational pre-organization can enhance the binding of a peptide ligand to its target (c.f. **100** vs. **101**).

We have already seen an example of conformational pre-organization delivering higher binding affinity (Section 5.2). Another example of the concept of conformational pre-organization is seen with thrombin inhibitor **99** (Figure 12). This molecule is disordered in solution, with the proline side chain interconverting between the C^4^-*endo* and C^4^-*exo* puckers; however, only the latter conformation is suitable for target binding [210]. In the fluorinated analog **100**, the required C^4^-*exo* pucker is pre-organized, and this leads to stronger target binding. Conversely, in the fluorinated analog **101**, the “wrong” C^4^-*endo* pucker is pre-organized, and this leads to weaker target binding.

## 7. Conclusions

Side chain-fluorinated amino acids offer fascinating and fruitful research opportunities. 

Synthetic chemistry underpins the field. A wide variety of methods for synthesizing side chain-fluorinated amino acids have now been established (outlined in Section 2.1 of this review), encompassing nucleophilic, electrophilic, metal-catalyzed, and photochemical fluorination methods. These synthetic advances have enabled the creation of structurally diverse amino acid targets, ranging from selectively fluorinated structures bearing one or a small number of fluorines on the side chain, all the way up to perfluorinated structures bearing a large number of fluorines. A range of methods for elaborating such fluorinated amino acids into peptides and proteins are also available (Section 2.2), including both chemical and biochemical strategies.

Fluorine is able to perform a variety of roles within amino acid side chains. The first role (described in Section 3 of this review) may be likened to that of a “spy”: fluorine enables the collection of information about the properties of biologically relevant molecules, through ^19^F-NMR or ^18^F-PET analysis. Fluorine’s second role (Section 4) is akin to that of a “tinker”: fluorine can repair some of the well-known pharmacokinetic problems associated with peptide-based drugs, including their permeability and their susceptibility to metabolism. Fluorine’s third role (Section 5) invites comparison with that of a “tailor”: fluorine can control the ways in which amino acids, peptides, and proteins fold. Fluorine’s fourth and final role (Section 6) may be likened to that of a “soldier”: fluorine can guide molecules to better hit their biological targets through both covalent and non-covalent means. 

In the future, it seems likely that research into side chain-fluorinated amino acids will continue to yield valuable outcomes. Two areas merit special mention. First, the concept of site-selective, late-stage protein fluorination [298] is an exciting development that could enable a greater variety of fluorinated proteins to be created for diverse applications. Second, it is noteworthy that although many side chain-fluorinated amino acids have been examined in this review, the examples that are *stereoselectively fluorinated* are relatively scarce and mostly limited to proline analogs; there seems to be an opportunity to further explore a wider variety of stereoselectively fluorinated amino acids for applications such as conformational control and enhancement of target-binding.
